# Anthropometric Indices of Giardia-Infected Under-Five Children Presenting with Moderate-to-Severe Diarrhea and Their Healthy Community Controls: Data from the Global Enteric Multicenter Study

**DOI:** 10.3390/children8121186

**Published:** 2021-12-15

**Authors:** Shamsun Nahar Shaima, Sumon Kumar Das, Shahnawaz Ahmed, Yasmin Jahan, Soroar Hossain Khan, Gazi Md. Salahuddin Mamun, Abu Sadat Mohammad Sayeem Bin Shahid, Irin Parvin, Tahmeed Ahmed, A. S. G. Faruque, Mohammod Jobayer Chisti

**Affiliations:** 1 Nutrition and Clinical Services Division, International Centre for Diarrhoeal Disease Research, Dhaka 1212, Bangladesh; shamsun.shaima@icddrb.org (S.N.S.); dr.yasminjahan@gmail.com (Y.J.); soroar@icddrb.org (S.H.K.); gazi.mamun@icddrb.org (G.M.S.M.); irin.parvin@icddrb.org (I.P.); tahmeed@icddrb.org (T.A.); gfaruque@icddrb.org (A.S.G.F.); chisti@icddrb.org (M.J.C.); 2 Menzies School of Health Research, Casuarina Campus, Charles Darwin University, Casuarina, Darwin, NT 0811, Australia; dassumonkumar1@gmail.com; 3 School of Health and Rehabilitation Science, University of Queensland, Brisbane, QLD 4067, Australia; shahnawaz.ahmed@uq.net.au

**Keywords:** giardiasis, *Giardia lamblia*, Global Enteric Multicenter Study, moderate-to-severe diarrhea, under-five children

## Abstract

Among all intestinal parasitosis, giardiasis has been reported to be associated with delayed growth in malnourished children under 5 living in low- and middle-income countries. Relevant data on the nutritional status of children aged 0–59 months presenting with moderate-to-severe diarrhea (MSD) and giardia infection were collected from sentinel health facilities of the Global Enteric Multicenter Study’s (GEMS) seven field settings, placed in diverse countries of Sub-Saharan Africa and South Asia between, December 2007 and February 2011. Then, this study analyzed a robust dataset of study participants (*n* = 22,569). Children having giardiasis with MSD constituted as cases (*n* = 1786), and those without MSD constituted as controls (*n* = 3470). Among the seven field sites, symptomatic giardiasis was 15% and 22% in Asian and African sites, respectively, whereas asymptomatic giardia infection (healthy without MSD) in Asian and African sites was 21.7% and 30.7%, respectively. Wasting and underweight were more frequently associated and stunting less often associated with symptomatic giardiasis (for all, *p* < 0.001). Symptomatic giardiasis had a significant association with worsening of nutritional status in under-five children. Improved socio-economic profile along with proper sanitation and hygienic practices are imperative to enhance child nutritional status, particularly in resource limited settings.

## 1. Introduction

Diarrhea and malnutrition are still leading public health alarms in under-five children, mostly in low- and middle-income countries. Researchers have indicated that children with recurrent attacks of diarrheal episodes are susceptible to malnutrition [1]. Globally, the most recent data that revealed <500,000 under-five deaths from diarrhea [2] are frequently affecting children from Sub-Saharan Africa and South Asia [1]. Several enteric pathogens have been implicated as important causes of such deaths, with only a few reviews using standardized methods that have elaborately mentioned the importance of all of these enteric pathogens [3] and the associated nutritional disorders. Giardiasis, caused by *Giardia lamblia*, is one of the parasitic diseases that mainly affect humans and many other mammals. It can colonize in the human intestine and lead to endothelial dysfunction, malabsorption of essential nutrients, causing nausea, diarrhea, abdominal distension, cramps, weight loss, anemia, and general weakness lasting from a few weeks to several months [4]. All these health disorders may even lead to death. Black et al. reported that chronic giardia infection may be asymptomatic [4], which also has a damaging effect on nutritional status. A study conducted in Malawi among 6-to-18-month-old children reported association between asymptomatic giardia infection and growth retardation, particularly stunting [5]. Another study that measured nutritional status of a cohort of children participating in the My Health project in six districts of three Cambodian provinces during their third follow-up round revealed that measures aiming at protecting from extensive exposures to animal feces can alleviate the burden of giardiasis and its penalties, like stunting [6]. Prevalence of *Giardia lamblia* among pre-school and school-going children had been reported to be 73.4% [7]. A 5.5% prevalence rate of *Giardia lamblia* had been documented in rural and urban populations in and around Union Territory of Chandigarh, India. The study indicated the highest prevalence rate among the slum dwellers (24.6%), while the most susceptible age-group was children aged less than five years (18%) [8]. The prevalence of *Giardia lamblia* in Southeast Asian countries, like Cambodia and mountainous northwest Vietnam, was 3.3% and 3.2%, respectively, due to a mixed infection with other parasites [9]. On the other hand, prevalence of *Giardia lamblia* in Far East Asian Countries, like Korea, was as low as 1.5%, which was associated with other parasitic infections [10]. Further, in China, students from kindergarten to university had a giardia prevalence of 6.08% with no other concomitant infections [7]. A study in the Gambia disclosed high prevalence of giardia in children with chronic diarrhea and malnutrition, and their infections did not respond to standard therapeutic measures [11]. Studies conducted in urban slum, Dhaka, revealed prevalence of giardia in diarrheal stool was 11.08% among pre-school children [12]. A study in urban Bangladesh mentioned the prevalence of *Giardia* *lamblia* infection in under-five children was 12.7%; however, 7.7% children were symptomatic, and 18% had asymptomatic infections [13]. Another case-control study conducted in Dhaka, Bangladesh, stated that among the 814 cases with community matched controls, giardia was more common in non-diarrheal controls compared to diarrheal children [14].

Global Enteric Multicenter Study (GEMS) intended to apprise the policy makers, researchers, and public health professionals with the comprehensive and most updated information regarding etiologic agents and the population-based burden of moderate-to-severe diarrhea (MSD) in 0–59-month-old children [1]. In this study, less than 5% of under-five children with MSD were infected with *Giardia lamblia,* and they presented with the typical syndrome of gastroenteritis [15].

There is a lack of data that examines the association of giardiasis (both in symptomatic diarrheal children and asymptomatic healthy control children) with the worsening nutritional status. Therefore, this study aimed to compare the anthropometric indices of giardia infected under-five children between those with MSD and their healthy community controls. 

## 2. Materials and Methods

### 2.1. Ethical Consideration

This multicenter study was approved by the Institutional Review Boards (IRBs) of the University of Maryland, Baltimore, MD, USA, and all the sites, including Bangladesh. IRB of Bangladesh was constituted with Research Review Committee and Ethical Review Committee whose project’s identification code was PR-2006-32, and date of approval was on 10 April 2006.

### 2.2. Study Site

The seven GEMS’s field settings (four urban and three rural) were established based on pre-set selection criteria in diverse countries of Sub-Saharan Africa (Kenya, Mali, Mozambique, and Gambia) and South Asia (Bangladesh, India, and Pakistan). Under-five children with MSD and their age-sex-community-matched healthy controls were concurrently enrolled into the study [16].

### 2.3. Study Design and Study Participants Enrollment Procedure

This three-year study followed a prospective, age-stratified, case-control-cohort design with a single household follow-up during 50–90 days after discharge [17]. Between December 2007 and February 2011, in every site of GEMS, children aged 0–59 months (0–11, 12–23, and 24–59 months); presenting with MSD; and meeting at least one of the following enrolment criteria: eyes sunken, skin turgor lost, prescribed or received intravenous rehydration therapy, requiring hospitalization, and distinct visible blood in stool were included in the GEMS. Nutritional indices (underweight, stunting, and wasting) were estimated following the World Health Organization’s (WHO) guidelines [18]. Controls of this study were matched to the cases by age, sex, and community. They were enrolled concurrently (within 7 days of the corresponding case enrollment). Caretakers of cases were interviewed at the sentinel health center (SHC) after their enrolment, whereas control children’s caretakers were subjected to interview at their household after their inclusion into the study. Linkages were made between study data and existing demographic surveillance system (DSS) databases, and data collection was also planned to include information from primary sources of DSS database. For the entire study period, the laboratory of each site of GEMS provided the diagnostic results to the clinicians working in the SHCs. Such information was used to improve case management strategies. From the database of GEMS, secondary relevant data were extracted for the present analysis.

### 2.4. Specimen Collection and Laboratory Procedure

Fresh stool specimens were collected during the enrolment of children following GEMS laboratory procedure protocol [16]. At least three grams of stool were collected from each participating child in the sentinel health facility.

Specimens were tested for the detection of comprehensive etiologic agents, including bacterial pathogens, protozoal agents, and viruses, following standard laboratory procedures. *Giardia lamblia* was diagnosed with commercially available enzyme-linked immunoassays (Tech Lab, Blacksburg, VA, USA) [19].

## 3. Statistical Analysis

For the present secondary data analysis, data representing all required variables were extracted from the GEMS database. Analysis of data was performed using SPSS, Windows (Version 20, Chicago, IL, USA). Epi Info (Version 7.0) aided in the typical 2/2 analysis to estimate unadjusted odds ratios. Chi-square (χ2) test appraised the significance of differences between categorical variables of interest. The strength of association reflected the measure of relationship between the dependent and independent variable by approximating odds ratio (OR) and 95% confidence interval (CIs). Principal component analysis (PCA) was computed, and the variables that were considered in PCA included construction materials of the wall, roof, and floor of the house and household assets, like radio, television, cell phone, and table. The wealth index was computed using PCA. The households were classified by quintiles reflecting socioeconomic profile: poor, lower middle, middle, upper middle, and rich. Variance inflation factor (VIF) intended to examine the multicollinearity status between independent variables before performing logistic regression. The VIF values were observed to be less than 2.0, and their mean was 1.17. Multivariate logistic regression analysis was undertaken to determine the factors that were significantly associated with symptomatic and asymptomatic giardia infection after adjusting for covariates. The model considered the cut-off points as 0.1 for *p*-value to prevent residual confounding in logistic regression [17]. *p*-Value < 0.05 was well-thought-out to replicate statistical significance.

## 4. Results

**Figure 1:**
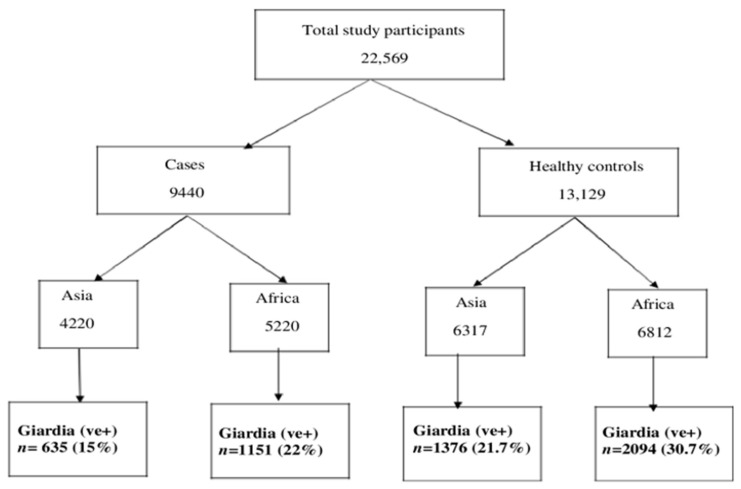
Study flow chart showing the distribution of cases and healthy controls in Asia and Africa.

**Figure 2:**
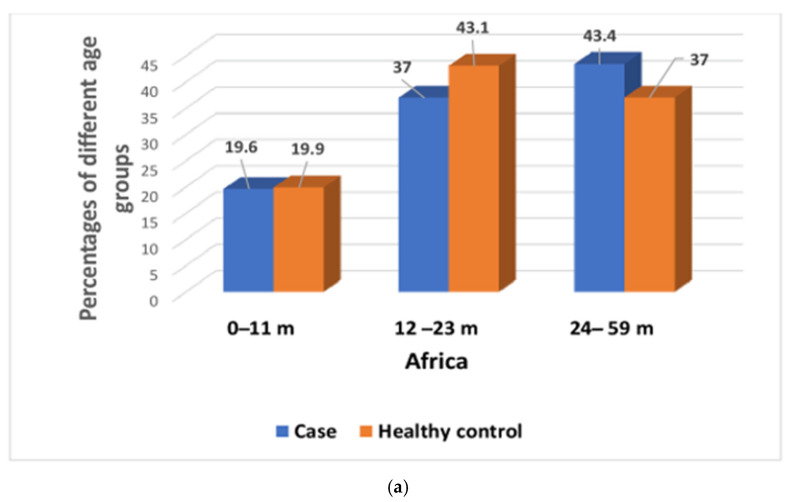

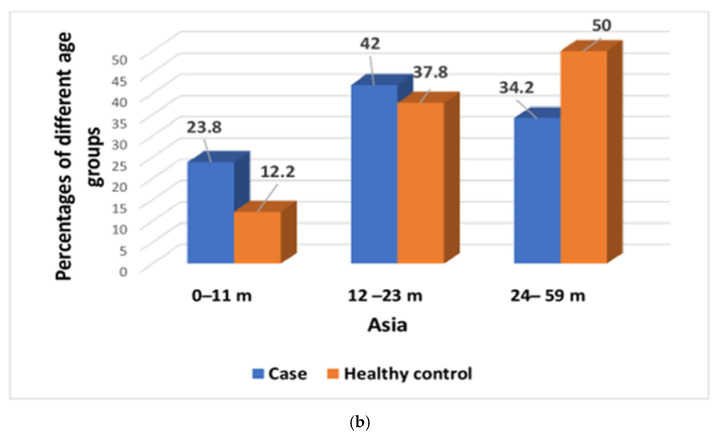
(**a**) Percentages of both symptomatic giardiasis and asymptomatic giardia infection among different age groups in Africa. (**b**) Percentages of both symptomatic giardiasis and asymptomatic giardia infection among different age groups in Asia.

**Figure 3:**
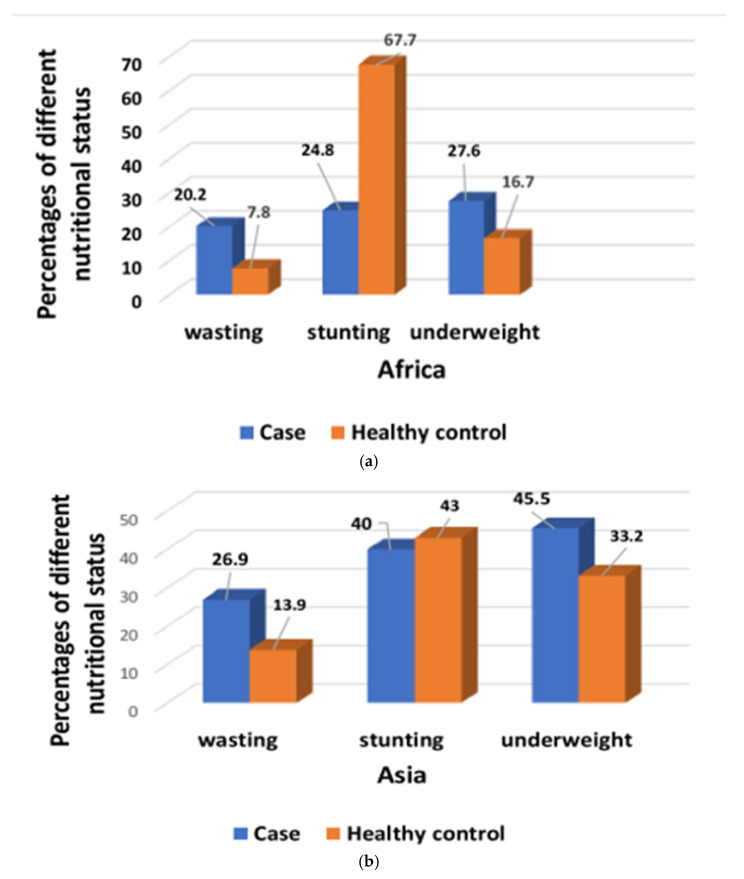
(**a**) Percentages of both symptomatic giardiasis and asymptomatic giardia infection among children with different nutritional status in Africa. (**b**) Percentages of both symptomatic giardiasis and asymptomatic giardia infection among children with different nutritional status in Asia.

**Figure 4:**
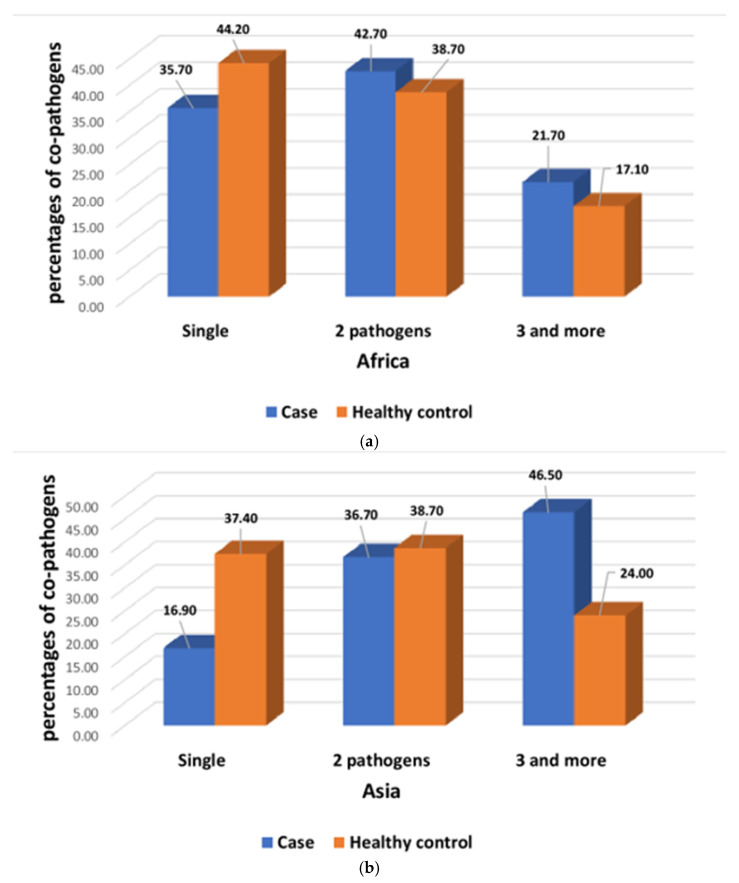
(**a**) Percentages of co-pathogens among both symptomatic giardiasis and asymptomatic giardia infection in Africa. (**b**) Percentages of co-pathogens among both symptomatic giardiasis and asymptomatic giardia infections in Asia.

A total of 22,569 participants were analyzed, of whom 9440 children had MSD, and 13,129 were asymptomatic, healthy children. Among the seven study field sites, 4220 participants belonged to the Asian sites, and 5220 participants were from the African sites. All of them had MSD, whereas 6317 and 6812 participants were healthy controls from the Asian and African sites, respectively (Figure 1). A total of 5256 (23.28%) participants had giardia infections among 22,569 study participants. In Asia, 15% study participants had symptomatic, and 21.7% had asymptomatic giardia infection, whereas in Africa, 22% of participants reported to have symptomatic, and another 30.7% had asymptomatic giardia infection. In African sites, children aged 24–59 months had higher percentages of symptomatic giardiasis than asymptomatic giardia infection (Figure 2a) and were significantly associated with symptomatic giardiasis (*p* < 0.001) compared to asymptomatic giardia infections (Table 1). In Asian sites, children aged 0–11 months were associated with symptomatic giardiasis compared to asymptomatic giardia infection (*p* < 0.001) (Table 2), and also percentages were higher among symptomatic giardiasis compared to asymptomatic giardia infection (Figure 2b). In terms of gender, it was comparable between both the groups. In case of socioeconomic status, in Africa, children from richer context were significantly more vulnerable to giardiasis (*p* = 0.002) (Table 1), whereas children from Asian settings were less frequently associated with giardiasis (*p* < 0.001) (Table 2). Hand-washing practice after defecation and after cleaning the bottom of child following defecation were associated with less chance of developing giardiasis (*p* = 0.001) in Asian sites (Table 2). In African site, percentages of wasting and underweight were higher in symptomatic giardiasis, whereas percentages of stunting was higher in asymptomatic giardia infection (Figure 3a), and the same observation was detected in Asian sites (Figure 3b). Wasting and underweight were significantly associated with symptomatic giardiasis in both African (Table 1) and Asian sites (*p* = 0.001) (Table 2). Participants from African sites were reported to be using water for their day-to-day activities mostly from deep tube wells (99.1%). In African children, usage of shallow tube well water was not associated with symptomatic giardiasis (Table 1), whereas in Asia, shallow-tube well users were 32% less likely to have symptomatic giardiasis (*p* = 0.027) (Table 2). In Figure 4a, percentages of single pathogen along with giardia are high in asymptomatic giardia infection, and two or more pathogens are associated with symptomatic giardiasis in Africa. Percentages are also higher in symptomatic giardiasis compared to asymptomatic giardia infection.

After adjusting by backward stepwise logistic regression, we observed that in African sites, giardiasis was less frequently associated with the children aged from 12–23 months and 24–59 months, whereas in Asian sites, symptomatic giardiasis was significantly associated with the both age groups than the reference group (0–11 months) (*p* = 0.001). Symptomatic giardiasis in Asian sites were more often significantly associated with children from poor socioeconomic status (*p* < 0.001). Besides, we found that giardiasis in African sites were significantly associated with the hygiene and sanitation practices, like hand washing before eating, prior to cooking, after defecation, and cleaning the bottom of the child following defecation (for all, *p* < 0.001). Among the children from each site, symptomatic giardiasis was more susceptible to have wasting and underweight, whereas it had less potential to be associated with stunting (*p* < 0.001) (Table 3).

## 5. Discussion

This study was designed to compare the anthropometric indices along with their socio-economic and demographic contexts among giardia infected under-five children with MSD and healthy controls across the seven sites of both Asia and Africa. Our study specified giardia as one of the commonly identified intestinal parasites in both the Asian and African sites. In this study, we also found that children from older age groups (12–59 months) were more often associated with giardiasis than children of younger age group (0–11 months) in Asian sites. Studies found that children aged 1–5 years or more were significantly associated with symptomatic giardiasis and more susceptible than those below one year of age [20,21]. This indicates that the infection transmission occurs frequently among those in their intermediate childhood, e.g., toddlers, especially when children commonly play in very close contact among themselves. Those children more exposed to outdoor activities than children of younger ages may disregard their hygiene and sanitation practices, which often likely to make them more exposed to infections that are prevalent among humans and a wide range of animals [22].

Besides, we found that symptomatic giardiasis among the children of poor socio-economic background were more often observed in Asian sites than African sites. One study conducted among children living in northeastern Brazil reported the higher prevalence of giardiasis (10.8%), where people more frequently had accompanying poverty, experiencing open defecation and using stored rainwater from the reservoirs for drinking. Studies also found that improved socio-economic status, proper sanitation, and hygienic practice may reduce the prevalence of symptomatic giardiasis and hospitalisation of symptomatic children as well [23]. Studies also reported that lack of hand washing and hygienic sanitation practices are associated with giardia infection [24,25], and our study also echoed with similar observations.

A study observation revealed objective evidence of declining transmission of enteric pathogens by handwashing with soap and hygienic sanitation interventions. Studies also reported that household-level platforms for handwashing, use of soapy water in the latrine and kitchen areas, and concomitant handwashing promotion can significantly reduce the giardia prevalence and excess infection [25]. Further, the handwashing intervention may cause lesser giardia transmission that has been observed to take place via hands of unhygienic caregivers’ hands and consumption of contaminated food, often indicated to be the universal routes of transmission [24]. Our study revealed that symptomatic giardiasis significantly influences children’s nutritional status specially causing wasting and underweight but negatively associated with stunting. In a MAL-ED study, Rogawski et al. reported a small decrease in length after three months and at age of 24 months in the case of community diarrheal episodes associated with bacteria and parasites. Substantial decrements in length (LAZ) at 24 months were associated with subclinical, non-diarrheal infections with giardia. The MAL-ED study had a birth-cohort design with anthropometric markers as the primary endpoint [26]. In our study, the data were collected as a part of GEMS, which followed case-control design with a subsequent follow-up visit at household level. Moreover, data were collected in sentinel health facilities at enrolment among MSD children. However, heterogeneity in study design, laboratory procedures [19,26], and data processing were observed between MAL-ED and GEMS studies. Our analysis segregated children with giardia and compared between children with MSD and healthy controls. In our study, stunting was observed significantly more often in healthy children with giardia than MSD children with giardiasis. Because of an apparent reverse causality, the present analysis, unlike wasting and underweight, observed a protective association between stunting and MSD episodes with giardia infection.

According to several studies, till now, evidences for an association between giardia infection and child growth is conflicting [27,28,29]. Giardia infection is associated with disordered villus architecture [30], which leads to an elevated lactulose/mannitol ratio (a marker of intestinal permeability) [31], and deficiencies of zinc and vitamin A [20,32], which are suggestive of structural and functional impairment of gut mucosa, causing inadequate nutrient uptake. A large population-based survey conducted in Tehran among school going children observed significant association between giardiasis and stunting and wasting [33]. Since determining factors are influencing growth at later ages more alarmingly, any intervention aiming to reduce burden of giardia infections during early life might cause optimal growth improvements in children beyond their infancy.

One of the major strengths of this study was quality laboratory performance ensuring standardized methods and use of large dataset. However, this study had few limitations. We did not have much information related to the use of both deep and shallow tube well water.

Moreover, we do not know whether study households were vulnerable to annual flooding or not, which sources of water were commonly used for household activities, the hygienic status of their toilets, as well as presence of garbage disposal area nearby the drinking water sources. Our findings were based only on children with MSD attending the selected sentinel health facilities; children who sought care without MSD and those not reporting to the sentinel health centers despite being recognized have MSD were not studied, and the study’s urban and rural settings might not represent the larger population living in their respective country.

## 6. Conclusions

The study may conclude that symptomatic giardiasis is related with worsening nutritional indices of under-five children compared to asymptomatic giardia infection. It was also observed that infection with giardiasis was more frequent among people from poor socio-economic contexts. These observations suggest that improvements in income, sanitation, and hygiene could possibly reduce the prevalence of giardiasis. To overcome this alarming scenario, more effective public health measures are needed to address the continuing public health concerns due to giardia infection and its consequences, including health education that may help to optimize personal hygiene and sanitary practices as well as improving general awareness of the parents/care-givers about parasitic infections, particularly in low-resource settings.

## Figures and Tables

**Table 1:** Comparison of characteristics between symptomatic giardiasis and asymptomatic giardia infection of under-five children in African sites.

Indicators	Cases Giardia (*n* = 1151) (%)	Healthy Controls Giardia (*n* = 2094) (%)	(Unadjusted) OR (95% CI)	*p*-Value
Age of the participants in months				
0–11	226 (19.6%)	417 (19.9%)	0.98 (0.82–1.17)	0.884
12–23	426 (37.0%)	902 (43.1%)	0.77 (0.66–0.90)	0.0001
24–59	499 (43.4%)	775 (37.0%)	1.30 (1.12–1.50)	0.001
Sex of the participants				
Female	537 (46.7%)	933 (44.6%)	1.08 (0.94–1.25)	0.265
Wealth quintile				
Rich	706 (61.3%)	1188 (56.8%)	1.20 (1.04–1.40)	0.012
Poor	445 (38.7%)	905 (43.2%)	0.83 (0.71–0.96)	0.012
Source of water				
Deep tube well	1141 (99.1%)	2077 (99.2%)	0.93 (0.43–2.04)	0.975
Shallow tube well	10 (0.9%)	17 (0.8%)	1.07 (0.48–2.34)	0.975
Use treated water				
Yes	246 (21.4%)	358 (17.1%)	1.31(1.09–1.57)	0.003
No	905 (78.6%)	1736 (82.9%)		
Toilet facility				
Sanitary	1086 (94.4%)	1964 (93.8%)	1.10 (0.81–1.50)	0.571
Non-sanitary	65 (5.6%)	130 (6.2%)	0.90 (0.66–1.22)	0.571
Hand-washing practices: Before eating				
Yes	1031 (89.6%)	1935 (92.4%)	0.70 (0.55–0.90)	0.007
No	120 (10.4%)	159 (7.6%)		
Before cooking				
Yes	624 (54.2%)	1519 (72.5%)	0.44 (0.38–0.52)	0.001
No	527 (45.8%)	575 (27.5%)		
After defecation				
Yes	804 (69.9%)	1660 (79.3%)	0.60 (0.51–0.71)	0.001
No	347 (30.1%)	434 (20.7%)		
After cleaning the bottom of child following defecation				
Yes	399 (34.7%)	1129 (53.9%)	0.45 (0.39–0.52)	0.001
No	752 (65.3%)	965 (46.1%)		
Nutritional status: Wasting				
Yes	232 (20.2%)	163 (7.8%)	2.99 (2.41–3.70)	0.001
No	918 (79.8%)	1927 (92.2%)		
Stunting				
Yes	285 (24.8%)	597 (67.7%)	0.82 (0.70–0.97)	0.024
No	865 (75.2%)	1496 (71.5%)		
Underweight				
Yes	318 (27.6%)	350 (16.7%)	1.90 (1.60–2.26)	0.001
No	833 (72.4%)	1743 (83.3%)		

*n*, number of subjects; OR, odds ratio; CI, confidence interval.

**Table 2:** Comparison of characteristics between symptomatic giardiasis and asymptomatic giardia infection of under five children in Asian sites.

Indicators	Cases Giardia (*n* = 635) (%)	Healthy Controls Giardia (*n* = 1376) (%)	(Unadjusted) OR (95% CI)	*p*-Value
Age of the participants in months				
0–11	151 (23.8%)	168 (12.2%)	2.24 (1.75–2.86)	<0.001
12–23	267 (42.0%)	520 (37.8%)	1.19 (0.98–1.44)	0.077
24–59	217 (34.2%)	688 (50.0%)	0.52 (0.43–0.63)	<0.001
Sex of the participants				
Female	286 (45.0%)	584 (42.4%)	1.11 (0.92–1.34)	0.296
Wealth quintile				
Rich	331 (52.1%)	838 (60.9%)	0.69 (0.58–0.85)	0.002
Poor	304 (47.9%)	538 (39.1%)	1.43 (1.18–1.73)	0.002
Source of water				
Deep tube well	585 (92.1%)	1222 (88.8%)	1.47 (1.05–2.05)	0.027
Shallow tube well	50 (7.9%)	154 (11.2%)	0.68 (0.48–0.95)	0.027
Use treated water				
Yes	204 (32.1%)	297 (21.6%)	1.71 (1.39–2.12)	0.001
No	431 (67.9%)	1079 (78.4%)		
Toilet facility				
Sanitary	611 (96.2%)	1306 (94.9%)	1.36 (0.85–2.19)	0.238
Non-sanitary	24 (3.8%)	70 (5.1%)	0.73 (0.45–1.17)	0.238
Hand-washing practices: Before eating				
Yes	491 (77.3%)	1037 (75.4%)	1.11 (0.89–1.39)	0.368
No	144 (22.7%)	339 (24.6%)		
Before cooking				
Yes	466 (73.4%)	990 (71.9%)	1.07 (0.87–1.32)	0.537
No	169 (26.6%)	386 (28.1%)		
After defecation				
Yes	474 (74.6%)	909 (66.1%)	1.51 (1.22–1.86)	0.001
No	161 (25.4%)	467 (33.9%)		
After cleaning the bottom of child following defecation				
Yes	362 (57.0%)	570 (41.4%)	1.87 (1.55–2.26)	0.001
No	273 (43.0%)	806 (58.6)		
Nutritional status: Wasting				
Yes	171 (26.9%)	191 (13.9%)	2.22 (1.81–2.89)	0.001
No	464 (73.1%)	1182 (86.1%)		
Stunting				
Yes	254 (40.0%)	591 (43.0%)	0.89 (0.73–1.07)	0.231
No	381 (60%)	783 (57.0%)		
Underweight				
Yes	289 (45.5%)	457 (33.2%)	1.68 (1.39–2.03)	0.001

*n*, number of subjects; OR, odds ratio; CI, confidence interval.

**Table 3:** Backward stepwise (conditional) logistic regression among symptomatic giardiasis and asymptomatic giardia infection of under-five children in both African and Asian sites.

Indicators Africa				Asia		
	Adjusted OR	95% CI	*p*-Value	Adjusted OR	95% CI	*p*-Value
Age group (0–11 m)	(Reference group)					
Age group (24–59 m)	0.79	(0.63–0.98)	0.026	2.41	(1.81–3.22)	<0.001
Age group (12–23 m)	0.70	(0.62–0.89)	<0.001	1.49	(1.20–1.86)	<0.001
Wealth quintile, poor	0.80	(0.69–0.93)	0.006	1.52	(1.23–1.88)	<0.001
Use treated water	-	-	-	0.66	(0.51–0.82)	<0.001
Handwashing before eating	1.42	(1.10–1.86)	0.008	-	-	-
Handwashing before cooking	2.11	(1.80–2.46)	<0.001	-	-	-
Handwashing after defecation	1.23	(1.03–1.48)	0.021	0.68	(0.53–0.84)	<0.001
Handwashing after cleaning the bottom of the child following defecation	2.06	(1.76–2.41)	<0.001	0.59	(0.48–0.72)	<0.001
Wasting	2.60	(1.96–3.46)	<0.001	1.69	(1.26–2.27)	<0.001
Stunting	0.66	(0.53–0.80)	<0.001	0.66	(0.51–0.84)	<0.001
underweight	1.57	(1.20–2.03)	<0.001	1.72	(1.30–2.30)	<0.001

OR, odds ratio; CI, confidence interval.

## Data Availability

The data set contained personal information of the study participants. Our institutional review board will not have the provision to disclose any kind of information. Thus, our policy is not to make available the data set in the manuscript, the supplemental files, or a public repository. However, data related to this manuscript are available upon request, and researchers who meet the criteria for access to confidential data may contact Ms. Armana Ahmed (aahmed@icddrb.org) to the research administration of icddr,b (http://www.icddrb.org/ accessed on 7 December 2021).

## References

[1] Muhsen K., Levine M.M. (2012). A systematic review and meta-analysis of the association between Giardia lamblia and endemic pediatric diarrhea in developing countries. Clin. Infect. Dis..

[2] Hug L., Dharrow D., Zhong K., You D. (2018). Levels and Trends in Child Mortality: Report 2018.

[3] Lanata C.F., Fischer-Walker C.L., Olascoaga A.C., Torres C.X., Aryee M.J., Black R.E., UNICEF (2013). Global causes of diarrheal disease mortality in children < 5 years of age: A systematic review. PLoS ONE.

[4] Srijan A., Wongstitwilairoong B., Pitarangsi C., Serichantalergs O., Fukuda C.D., Bodhidatta L., Mason C.J. (2005). Re-evaluation of commercially available enzyme-linked immunosorbent assay for the detection of Giardia lamblia and *Cryptosporidium* spp from stool specimens. Southeast Asian J. Trop. Med. Public Health.

[5] Lehto K.M., Fan Y.M., Oikarinen S., Nurminen N., Hallamaa L., Juuti R., Mangani C., Maleta K., Hyöty H., Ashorn P. (2019). Presence of Giardia lamblia in stools of six-to 18-month old asymptomatic Malawians is associated with children’s growth failure. Acta Paediatr..

[6] Caron Y., Hong R., Gauthier L., Laillou A., Wieringa F.T., Berger J., Poirot E. (2018). Stunting, beyond acute diarrhoea: Giardia duodenalis, in Cambodia. Nutrients.

[7] Dib H.H., Lu S.Q., Wen S.F. (2008). Prevalence of Giardia lamblia with or without diarrhea in South East, South East Asia and the Far East. Parasitol. Res..

[8] Khurana S., Aggarwal A., Malla N. (2005). Comparative analysis of intestinal parasitic infections in slum, rural and urban populations in and around union Territory, Chandigarh. J. Commun. Dis..

[9] Suman M., Alam M., Pun S., Khair A., Ahmed S., Uchida R. (2011). Prevalence of Giardia lamblia infection in children and calves in Bangladesh. Bangladesh J. Vet. Med..

[10] Guk S.-M., Seo M., Park Y.-K., Oh M.-D., Choe K.-W., Kim J.-L., Choi M.-H., Hong S.-T., Chai J.-Y. (2005). Parasitic infections in HIV-infected patients who visited Seoul National University Hospital during the period 1995–2003. Korean J. Parasitol..

[11] Kotloff K.L., Nataro J.P., Blackwelder W.C., Nasrin D., Farag T.H., Panchalingam S., Wu Y., Sow S.O., Sur D., Breiman R.F. (2013). Burden and aetiology of diarrhoeal disease in infants and young children in developing countries (the Global Enteric Multicenter Study, GEMS): A prospective, case-control study. Lancet.

[12] Haque R., Mondal D., Kirkpatrick B.D., Akther S., Farr B.M., Sack R.B., Petri W.A. (2003). Epidemiologic and clinical characteristics of acute diarrhea with emphasis on Entamoeba histolytica infections in preschool children in an urban slum of Dhaka, Bangladesh. Am. J. Trop. Med. Hyg..

[13] Haque R., Roy S., Kabir M., Stroup S.E., Mondal D., Houpt E.R. (2005). Giardia assemblage A infection and diarrhea in Bangladesh. J. Infect. Dis..

[14] Albert M.J., Faruque A., Faruque S., Sack R., Mahalanabis D. (1999). Case-control study of enteropathogens associated with childhood diarrhea in Dhaka, Bangladesh. J. Clin. Microbiol..

[15] Levine M.M., Kotloff K.L., Nataro J.P., Muhsen K. (2012). The global enteric multicenter study (GEMS): Impetus, rationale, and genesis. Clin. Infect. Dis..

[16] Farag T.H., Nasrin D., Wu Y., Muhsen K., Blackwelder W.C., Sommerfelt H., Panchalingam S., Nataro J.P., Kotloff K.L., Levine M.M. (2012). Some epidemiologic, clinical, microbiologic, and organizational assumptions that influenced the design and performance of the Global Enteric Multicenter Study (GEMS). Clin. Infect. Dis..

[17] Kotloff K.L., Blackwelder W.C., Nasrin D., Nataro J.P., Farag T.H., van Eijk A., Adegbola R.A., Alonso P.L., Breiman R.F., Golam Faruque A.S. (2012). The Global Enteric Multicenter Study (GEMS) of diarrheal disease in infants and young children in developing countries: Epidemiologic and clinical methods of the case/control study. Clin. Infect. Dis..

[18] World Health Organization (2006). WHO Child Growth Standards: Length/Height-for-Age, Weight-for-Age, Weight-for-Length, Weight-for-Height and Body Mass Index-for-Age: Methods and Development.

[19] Panchalingam S., Antonio M., Hossain A., Mandomando I., Ochieng B., Oundo J., Ramamurthy T., Tamboura B., Zaidi A.K., Petri W. (2012). Diagnostic microbiologic methods in the GEMS-1 case/control study. Clin. Infect. Dis..

[20] Abou-Shady O., El Raziky M.S., Zaki M.M., Mohamed R.K. (2011). Impact of Giardia lamblia on growth, serum levels of zinc, copper, and iron in Egyptian children. Biol. Trace Elem. Res..

[21] Botero-Garcés J.H., García-Montoya G.M., Grisales-Patiño D., Aguirre-Acevedo D.C., Álvarez-Uribe M.C. (2009). Giardia intestinalis and nutritional status in children participating in the complementary nutrition program, Antioquia, Colombia, May to October 2006. Rev. Inst. Med. Trop. São Paulo.

[22] Lass A., Karanis P., Korzeniewski K. (2017). First detection and genotyping of Giardia intestinalis in stool samples collected from children in Ghazni Province, eastern Afghanistan and evaluation of the PCR assay in formalin-fixed specimens. Parasitol. Res..

[23] Bello J., Núñez F., González O., Fernández R., Almirall P., Escobedo A. (2011). Risk factors for Giardia infection among hospitalized children in Cuba. Ann. Trop. Med. Parasitol..

[24] Bartelt L.A., Sartor R.B. (2015). Advances in understanding Giardia: Determinants and mechanisms of chronic sequelae. F1000prime Rep..

[25] Ashraf S., Nizame F.A., Islam M., Dutta N.C., Yeasmin D., Akhter S., Abedin J., Winch P.J., Ram P.K., Unicomb L. (2017). Nonrandomized trial of feasibility and acceptability of strategies for promotion of soapy water as a handwashing agent in rural Bangladesh. Am. J. Trop. Med. Hyg..

[26] Rogawski E.T., Liu J., Platts-Mills J.A., Kabir F., Lertsethtakarn P., Siguas M., Khan S.S., Praharaj I., Murei A., Nshama R. (2018). Use of quantitative molecular diagnostic methods to investigate the effect of enteropathogen infections on linear growth in children in low-resource settings: Longitudinal analysis of results from the MAL-ED cohort study. Lancet Glob. Health.

[27] Boeke C.E., Mora-Plazas M., Forero Y., Villamor E. (2010). Intestinal protozoan infections in relation to nutritional status and gastrointestinal morbidity in Colombian school children. J. Trop. Pediatrics.

[28] Ajjampur S., Koshy B., Venkataramani M., Sarkar R., Joseph A., Jacob K., Ward H., Kang G. (2011). Effect of cryptosporidial and giardial diarrhoea on social maturity, intelligence and physical growth in children in a semi-urban slum in south India. Ann. Trop. Paediatr..

[29] Centeno-Lima S., Rosado-Marques V., Ferreira F., Rodrigues R., Indeque B., Camará I., De Sousa B., Aguiar P., Nunes B., Ferrinho P. (2013). Giardia duodenalis and chronic malnutrition in children under five from a rural area of Guinea-Bissau. Acta Med. Port..

[30] Koot B.G., ten Kate F.J., Juffrie M., Rosalina I., Taminiau J.J., Benninga M.A. (2009). Does Giardia lamblia cause villous atrophy in children?: A retrospective cohort study of the histological abnormalities in giardiasis. J. Pediatric Gastroenterol. Nutr..

[31] Denno D.M., VanBuskirk K., Nelson Z.C., Musser C.A., Hay Burgess D.C., Tarr P.I. (2014). Use of the lactulose to mannitol ratio to evaluate childhood environmental enteric dysfunction: A systematic review. Clin. Infect. Dis..

[32] Astiazaran-Garcia H., Lopez-Teros V., Valencia M.E., Vazquez-Ortiz F., Sotelo-Cruz N., Quihui-Cota L. (2010). Giardia lamblia infection and its implications for vitamin A liver stores in school children. Ann. Nutr. Metab..

[33] Nematian J., Gholamrezanezhad A., Nematian E. (2008). Giardiasis and other intestinal parasitic infections in relation to anthropometric indicators of malnutrition: A large, population-based survey of schoolchildren in Tehran. Ann. Trop. Med. Parasitol..

